# Urate-lowering therapy modifies associations between urinary urate and kidney outcome

**DOI:** 10.1080/0886022X.2026.2700810

**Published:** 2026-08-24

**Authors:** Tomoaki Takata, Yukari Mae, Shotaro Hoi, Naoyuki Otani, Takaaki Sugihara, Ichiro Hisatome, Hajime Isomoto

**Affiliations:** ^a^ Division of Gastroenterology and Nephrology, Faculty of Medicine, Tottori University, Yonago, Japan; ^b^ Department of Cardiology, Dokkyo Medical University, Nikko Medical Center, Nikko, Japan; ^c^ Department of Cardiology, NHO Yonago Medical Center, Yonago, Japan; ^d^ Division of Pathobiological Science and Technology, Major in Clinical Laboratory Science, School of Health Science, Faculty of Medicine, Tottori University, Yonago, Japan; ^e^ Department of Cardiology, Matsue City Hospital, Matsue, Japan

**Keywords:** FEUA, ULT, urate excretion, uric acid, UUCR

## Abstract

**Background:**

Serum uric acid is associated with chronic kidney disease (CKD) progression; however, causality remains uncertain. Although renal urate handling, rather than merely hyperuricemia, may dominate kidney outcomes, the prognostic value of urinary urate indices and the potential modifying effect of urate-lowering therapy (ULT) remain unclear.

**Methods:**

This retrospective study included patients with an estimated glomerular filtration rate (eGFR) of 15–60 mL/min/1.73m^2^. Exposures were fractional excretion of uric acid (FEUA) and the urinary uric acid-to-creatinine ratio (UUCR). Major adverse kidney event (MAKE) was defined as ≥40% decline in eGFR, initiation of kidney replacement therapy, or death. Associations between FEUA and MAKE, and the modifying effect of ULT were evaluated using Kaplan–Meier analyses and multivariable Cox proportional hazards models, including stratification by baseline ULT.

**Results:**

Among 551 patients (244 receiving ULT), 117 experienced MAKE over a median follow-up of 2.3 years. In the adjusted analysis, FEUA <5.5% was associated with a higher overall risk (hazard ratio [HR], 3.56; 95% CI, 1.96–6.48), with a stronger association in the no-ULT group (HR, 11.59; 95% CI, 3.80–35.31). Conversely, FEUA was not associated with the outcome within the ULT group. FEUA × ULT interaction was significant, indicating that the prognostic meaning of FEUA depends on concomitant ULT.

C**onclusion:** Reduced urinary urate excretion was associated with a higher risk for MAKE. ULT modifies the prognostic impact of FEUA, underscoring the clinical relevance of renal urate handling when assessing kidney risk in CKD and suggesting that pharmacological urate-lowering may modify this risk.

## PLAIN LANGUAGE SUMMARY

Uric acid is usually assessed by its level in the blood. However, the serum urate level alone does not show how the kidneys handle it. In this study of 551 patients with chronic kidney disease, we assessed how much and how efficiently uric acid is excreted by the kidneys using two urinary measures of uric acid handling: the fractional excretion of uric acid and the urinary uric acid-to-creatinine ratio. Low values were associated with a greater risk of substantial kidney function decline, kidney replacement therapy, or death, particularly among patients not receiving urate-lowering therapy. Among treated patients, these relationships were different and less clear. These findings suggest that urinary urate assessment may help identify patients with kidney disease who are at higher risk.

## KEY MESSAGES

**What is known:**

Reduced urinary urate excretion has been associated with a higher risk of chronic kidney disease progression, but whether this association differs according to urate-lowering therapy status remains uncertain.

**This study adds:**

Low fractional excretion of uric acid and low urinary uric acid-to-creatinine ratio were independently associated with adverse kidney outcomes, mainly in patients not receiving urate-lowering therapy, and baseline treatment significantly modified this association.

**Potential impact:**

Urinary urate indices, interpreted together with urate-lowering therapy status and kidney function, may help identify patients with chronic kidney disease who are at higher risk of progression.

## Introduction

Hyperuricemia (HUA) is common among patients with chronic kidney disease (CKD) and has long been implicated in CKD development and progression [1,2]. Large cohort studies and meta-analyses have consistently shown that higher serum urate levels are associated with an increased risk of CKD onset and progression [3–6]. However, whether this association is causal remains controversial. Notably, several studies have reported that the association attenuates after adjustment for confounders [7–10]. Mendelian randomization analyses have not established a causal link [11], and randomized controlled trials of xanthine oxidase inhibitors (XOIs) have not revealed clear renal benefits [12–14]. These findings raise the question of whether serum urate is a mediator of kidney injury or merely a marker of underlying risk [15].

Recent studies have highlighted intrarenal urate handling as a potential determinant of kidney injury, suggesting that renal urate dynamics rather than hyperuricemia per se, may play a key role in renal pathophysiology. Experimental studies have shown that urate reabsorbed in the proximal tubule can trigger oxidative stress and inflammatory signaling in tubular epithelial cells, leading to interstitial fibrosis and kidney injury [16–19]. HUA arises from heterogeneous mechanisms, including overproduction, reduced extra-renal excretion, and impaired renal excretion [20,21]. Reduced renal urate excretion could be further classified into two phenotypes: glomerular under-filtration and tubular over-reabsorption [22,23], and these subtypes may differentially influence kidney outcomes. Nevertheless, evidence linking these subtypes to long-term kidney outcomes remains limited. Furthermore, few studies have examined the association between urinary urate excretion and kidney outcomes while accounting for urate-lowering therapy (ULT), which may influence urinary urate indices. Therefore, we aimed to investigate the association between urinary urate excretion and kidney outcomes, with particular attention to potential effect modification by ULT.

## Methods

### Study population

This retrospective cohort study included patients who visited the Department of Nephrology at Tottori University Hospital between January 2018 and December 2023. Eligible patients were aged ≥ 20 years, had a baseline estimated glomerular filtration rate (eGFR) of 15–60 mL/min/1.73m^2^, and had available data on urinary uric acid measurements. Patients with hypouricemia (baseline serum uric acid levels <3.0 mg/dL), acute kidney injury, or a history of dialysis or kidney transplantation were excluded. Participants were followed up from the date of the first available urinary uric acid measurement until the occurrence of the composite outcome, initiation or change of any ULT, loss to follow-up, or the end of the study period (September 30, 2025), whichever came first. The Institutional Review Boards of Tottori University Hospital approved this study (approval number: 23A079) and waived the requirement for informed consent because of the retrospective study design. This study was conducted in accordance with the Declaration of Helsinki.

### Data collection

Patient characteristics, medications, and laboratory data were obtained retrospectively from the electronic medical records. Variables included age, sex, body mass index (BMI), systolic and diastolic blood pressure, and history of diabetes mellitus, hypertension, cardiovascular disease, or gout. Medication data included the use of loop and thiazide diuretics, angiotensin-converting enzyme inhibitors, angiotensin II receptor blockers, sodium–glucose cotransporter 2 inhibitors, statins, fibrates, XOIs, and uricosuric agents. We recorded the dates of initiation or change of XOIs and uricosuric agents. Laboratory parameters included serum creatinine, uric acid, albumin, and C-reactive protein levels, as well as urinary pH, creatinine, uric acid, protein, N-acetylglucosamine (NAG), and β2-microglobulin (β2MG). Aciduria was defined as urine pH < 6.0. The eGFR was calculated from the serum creatinine levels using the Japanese equation [24].

### Outcome and exposures

The major adverse kidney event (MAKE) was defined as *a* ≥ 40% decline in eGFR from baseline, initiation of kidney replacement therapy, or death [25–27]. The primary exposure variable was the baseline fractional excretion of uric acid (FEUA) calculated as:

$$\text{FEUA}\left(\text{\%}\right)=\frac{\text{Urine}\;\text{UA}\;\left(\frac{\text{mg}}{\text{dL}}\right)\times \text{Serum}\;\text{Cr}\left(\frac{\text{mg}}{\text{dL}}\right)}{\text{Serum}\;\text{UA}\;\left(\frac{\text{mg}}{\text{dL}}\right)\times \text{Urine}\;\text{Cr}\left(\frac{\text{mg}}{\text{dL}}\right)}\times 100$$

To evaluate associations across levels of urinary urate excretion, patients were categorized by FEUA as <5.5%, 5.5–11.0% (reference range), and >11.0% based on prior reports [20,28]. Urinary uric acid–to–creatinine ratio (UUCR) was calculated as a secondary variable and categorized as <0.25, 0.25–0.50 (reference range), and >0.50 [29–31].

### Statistical analysis

Continuous variables are presented as means ± SD or medians (interquartile ranges [IQR]), and categorical variables as n (%). Kaplan–Meier curves were compared by log–rank tests. Associations between FEUA/UUCR categories and kidney outcome were evaluated using Cox proportional hazards models with multivariable adjustment (age, sex, BMI, baseline eGFR, systolic blood pressure, diabetes mellitus, proteinuria, urinary NAG–to–creatinine ratio, urinary β2MG–to–creatinine ratio, serum uric acid, serum albumin, and concomitant medications). Multivariable models were based on complete cases. Effect modification by ULT was analyzed using stratified analyses and FEUA × ULT interaction terms. Two-sided *p* < 0.05 was considered significant. Analyses used StatFlex (version 7.0 for Windows; Artec, Osaka, Japan), GraphPad Prism (7.0 for Windows, GraphPad Software, San Diego, CA, USA), or R software (version 4.5.2, R Foundation for Statistical Computing, Vienna, Austria).

## Results

### Baseline characteristics

**Figure 1.**
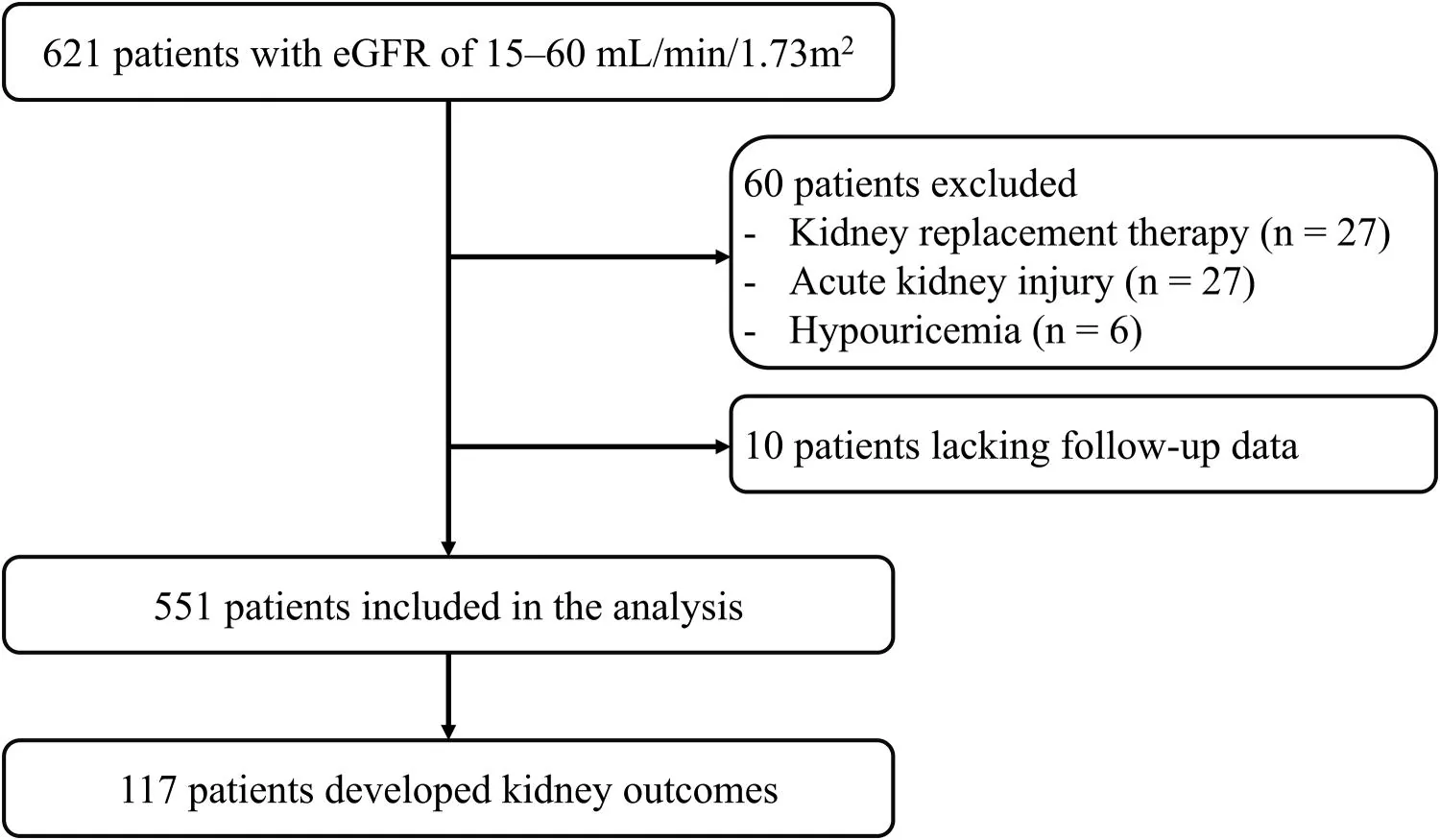
Study flow diagram showing patient selection. Among the 621 eligible patients, 60 were excluded (27 with a history of kidney replacement therapy, 27 with acute kidney injury, and six with hypouricemia). An additional 10 patients were excluded because of missing follow-up data, leaving 551 patients for the final analysis (307 in the no-ULT group and 244 in the ULT group). ULT, urate–lowering therapy.

Among 621 eligible patients, 70 were excluded, leaving 551 patients for the final analysis (Figure 1). At baseline, 244 patients received ULT (ULT group) and 307 did not (no–ULT group). Among the 244 patients receiving ULT, 240 (98.4%) were treated with an XOI (febuxostat in 204, allopurinol in 30, and topiroxostat in 6), and four (1.6%) received a uricosuric agent (benzbromarone in 3 and dotinurad in 1).

Patients’ baseline characteristics are summarized in Table 1. Indices of urinary urate excretion were significantly higher in the no–ULT group than in the ULT group (Table 1 and Supplemental Fig. S1). The median FEUA was 8.3% (IQR, 6.6–10.6) in the no–ULT group and 5.2% (IQR, 4.7–7.3) in the ULT group (*p* < 0.001). The median UUCR was 0.42 g/gCr (IQR, 0.33–0.50) and 0.20 g/gCr (IQR, 0.15–0.28) (*p* < 0.001).

**Table 1.** Baseline characteristics of the patients.

			ULTs		
	Total	Missing data	(-)	(+)	p value
Number	551		307	244	
Age, years	70 ± 13	0	69 ± 13	70 ± 13	0.41
Male, n (%)	372 (68%)	0	181 (59%)	191 (78%)	<0.001
BMI, kg/m^2^	24.0 ± 4.0	1 (< 1%)	23.8 ± 3.9	24.2 ± 4.1	0.27
Systolic blood pressure, mmHg	137 ± 23	0	139 ± 23	134 ± 22	0.015
Diastolic blood pressure, mmHg	79 ± 15	0	80 ± 15	77 ± 15	0.010
Diabetes mellitus, *n* (%)	160 (29%)	0	99 (32%)	61 (25%)	0.063
Hypertension, *n* (%)	438 (79%)	0	225 (73%)	213 (87%)	<0.001
Cardiovascular disease, *n* (%)	163 (30%)	0	73 (24%)	90 (37%)	<0.001
Gout, *n* (%)	18 (3%)	0	2 (1%)	16 (7%)	<0.001
Loop diuretics, *n* (%)	85 (15%)	0	31 (10%)	54 (22%)	<0.001
Thiazide diuretics, *n* (%)	51 (9%)	0	22 (7%)	29 (12%)	0.30
RAS blockades, *n* (%)	323 (59%)	0	163 (53%)	160 (66%)	0.003
Statins, *n* (%)	221 (40%)	0	113 (37%)	108 (44%)	0.076
SGLT2 inhibitors, *n* (%)	25 (5%)	0	16 (5%)	9 (4%)	0.39
Albumin, g/dL	3.9 ± 0.6	2 (< 1%)	3.8 ± 0.6	3.9 ± 0.5	0.30
Creatinine, mg/dL	1.4 (1.1–1.8)	0	1.2 (1.0–1.6)	1.6 (1.3–2.2)	<0.001
eGFR, mL/min/1.73 m^2^	37.0 ± 12.1	0	40.4 ± 11.5	32.7 ± 11.5	<0.001
Uric acid, mg/dL	6.5 ± 1.5	0	6.6 ± 1.5	6.3 ± 1.4	0.062
C–reactive protein, mg/dL	0.1 (0.0–0.3)	185 (34%)	0.1 (0.0–0.3)	0.2 (0.1–0.4)	0.039
Acidic urine (pH < 6.0), *n* (%)	233 (42%)	0	119 (39%)	114 (47%)	0.060
UPCR, g/gCr	0.4 (0.0–1.5)	6 (1%)	0.3 (0.1–1.6)	0.4 (0.1–1.3)	0.94
Urinary NAG–to–Cr ratio, U/gCr	10.7 (6.3–17.2)	97 (18%)	11.2 (6.2–17.6)	10.2 (6.6–16.9)	0.84
Urinary β2MG–to–Cr ratio, µg/gCr	541 (143–3153)	78 (14%)	560 (182–2948)	486 (97–4681)	0.24
FEUA, %	7.0 (5.0–9.4)	0	8.3 (6.6–10.6)	5.2 (4.0–7.3)	<0.001
UUCR, g/gCr	0.33 (0.20–0.44)	0	0.42 (0.33–0.50)	0.20 (0.15–0.28)	<0.001

BMI, body mass index; RAS, renin–angiotensin system; SGLT2, sodium–glucose cotransporter 2; eGFR, estimated glomerular filtration rate; UPCR, urinary protein–to–creatinine ratio; FEUA, fractional excretion of uric acid; NAG, N-acetylglucosamine; β2MG, beta 2–microglobulin; UUCR, urinary uric acid–to–creatinine ratio; ULT, urate–lowering theapy; XOI, xanthine oxidase inhibitor. Aciduria–urine pH < 6.0.

Baseline characteristics stratified by FEUA are summarized for the no–ULT and ULT groups in Supplemental Tables S1 and S2, respectively. In the no–ULT group, clinical characteristics were generally comparable across FEUA categories. However, patients with low FEUA had higher serum uric acid and lower UUCR than the other groups, whereas those with high FEUA had the lowest eGFR and the highest urinary β2MG (Supplemental Table S1). In the ULT group, FEUA categories were also associated with differences in eGFR and UUCR: patients with high FEUA had the lowest eGFR, the highest urinary β2MG and UUCR, and the lowest serum uric acid levels (Supplemental Table S2). In the overall cohort, FEUA was strongly correlated with UUCR (Spearman’s rho = 0.708, *p* < 0.001), whereas the correlation between FEUA and eGFR was weak (Spearman’s rho = −0.113, *p* = 0.008) (Supplemental Fig. S2). Similar patterns were observed in the no-ULT and ULT groups.

**Table 2.** Cox proportional hazards models stratified by baseline ULT use.

Subgroup	FEUA category	Adjusted HR (95% CI)	p value
Overall	< 5.5%	3.56 (1.96–6.48)	< 0.001
(*n* = 551)	5.5-11.0%	Reference	―
	> 11.0%	0.66 (0.32–1.35)	0.25
No-ULT group	< 5.5%	11.59 (3.80–35.31)	< 0.001
(*n* = 307)	5.5-11.0%	Reference	―
	> 11.0%	0.49 (0.14–1.70)	0.26
ULT group	< 5.5%	1.92 (0.85–4.34)	0.12
(*n* = 244)	5.5-11.0%	Reference	―
	> 11.0%	1.35 (0.52–3.52)	0.54

ULT, urate lowering therapy; FEUA, fractional excretion of uric acid; HR, hazard ratio; CI, confidence interval.

### Association between FEUA and kidney outcome

During a median follow-up of 2.3 years (IQR, 1.2–3.9), 117 patients (21.2%) achieved the composite outcome, including 14 deaths and seven initiations of kidney replacement therapy. The overall incidence rate of kidney outcome was 7.5 events per 100 person–years.

**Figure 2.**
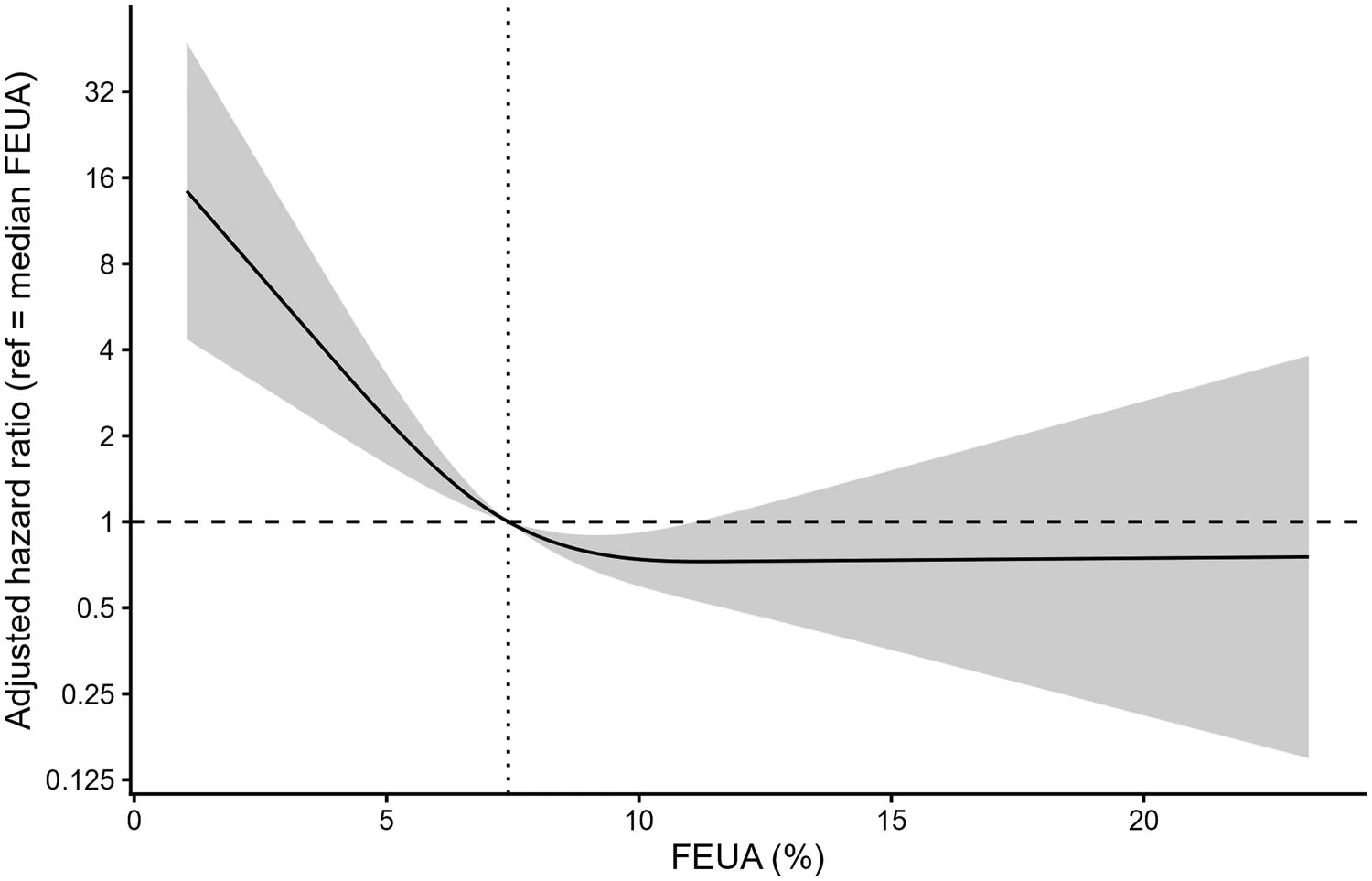
Restricted cubic spline analyses of FEUA in relation to composite outcome. Restricted cubic spline plots showing the adjusted association between FEUA (%) and the risk of the composite outcome. The solid line indicates the adjusted hazard ratio from the Cox model, and the shaded area indicates the 95% confidence interval. Hazard ratios are presented relative to the median value of each exposure (HR = 1, horizontal dashed line). Vertical dotted lines indicate the median exposure values. Models were adjusted for age, sex, body mass index, baseline eGFR, systolic blood pressure, diabetes mellitus, proteinuria, urinary NAG–to–creatinine ratio, urinary β2MG–to–creatinine ratio, serum uric acid, serum albumin, and concomitant medications, including urate-lowering therapy. FEUA, fractional excretion of uric acid.

**Figure 3.**
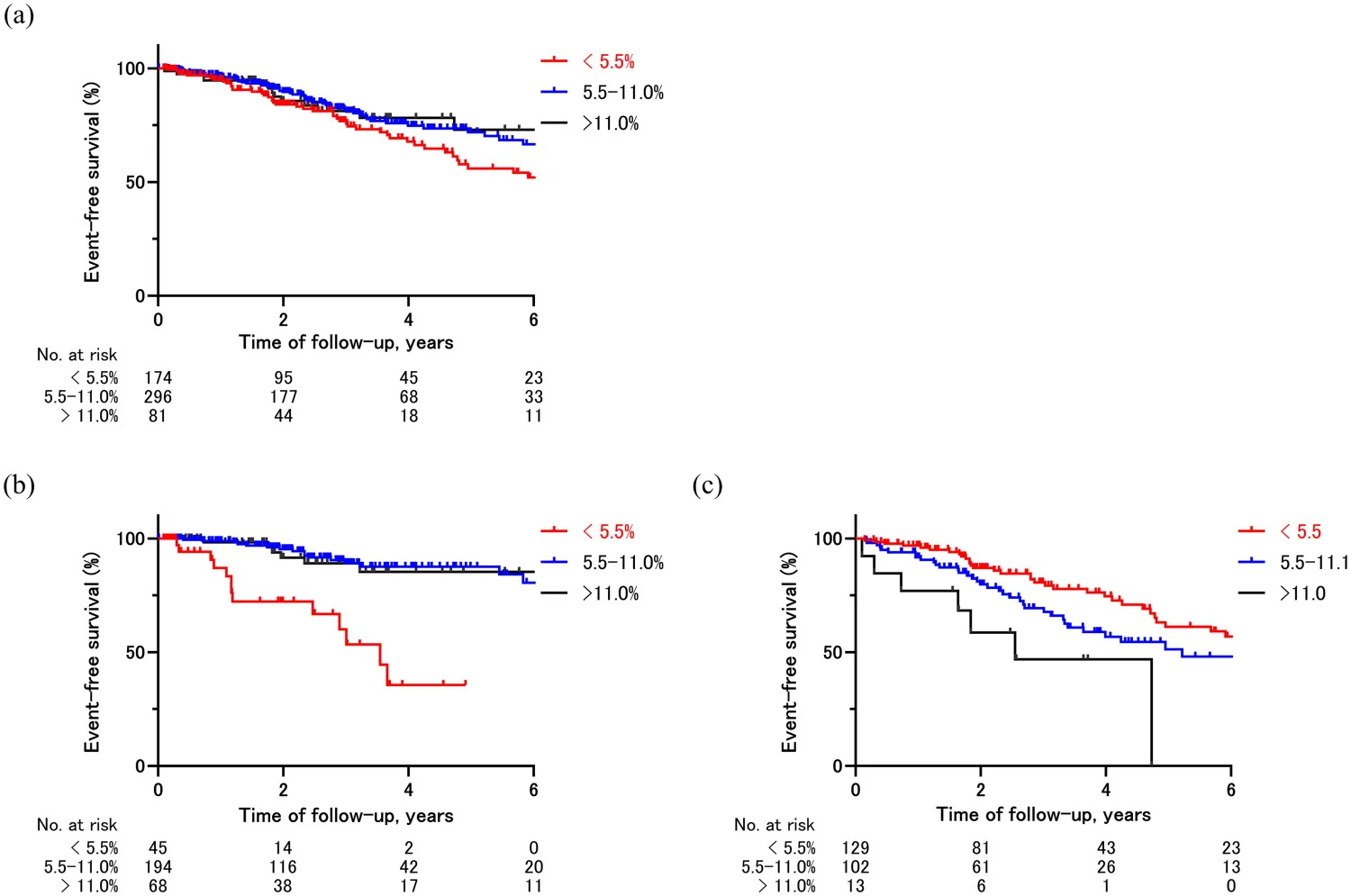
Kaplan–Meier curves for the composite outcome based on FEUA categories. Kaplan–Meier curves for the composite outcome stratified by baseline FEUA categories: <5.5%, 5.5–11.0%, and >11.0%. (a) Overall cohort (b) Patients not receiving urate–lowering therapy. (c) Patients receiving urate–lowering therapy at baseline. Comparisons were performed using the log-rank test. FEUA, fractional excretion of uric acid.

Restricted cubic spline analysis in the overall cohort showed that the adjusted hazard increased steeply as FEUA decreased below the median, whereas the estimated hazard ratio remained relatively flat at higher FEUA values (Figure 2). Next, we performed Kaplan–Meier curve analysis based on FEUA categories. In the overall cohort, patients with low FEUA had poorer kidney survival than those with higher FEUA (log-rank *p* = 0.062). When stratified by baseline ULT use, the pattern differed between groups (Figure 3). In the no–ULT group, patients with FEUA <5.5% had poorer kidney survival (log–rank *p* < 0.001). In the ULT group, those with FEUA >11.0% showed poorer unadjusted kidney survival (log–rank *p* = 0.004).

Subsequently, we evaluated the association between FEUA categories and the risk of outcome using Cox proportional hazards models (Table 2). In the overall cohort, low FEUA was significantly associated with the risk of the composite outcome. Using FEUA 5.5–11.0% as the reference, the HR for the low FEUA group was 3.56 (95% CI, 1.96–6.48), and that for the high FEUA group was 0.66 (95% CI, 0.32–1.35). Notably, the association between FEUA and the composite outcome differed by ULT use. In the no–ULT group, FEUA <5.5% was strongly and independently associated with the outcome (adjusted HR, 11.59; 95% CI, 3.80–35.31), whereas FEUA >11.0% was not significantly associated with the outcome (adjusted HR, 0.49; 95% CI, 0.14–1.70). In the ULT group, neither low nor high FEUA was associated with the composite outcome (adjusted HR, 1.92; 95% CI, 0.85–4.34 and 1.35; 95% CI, 0.52–3.52, respectively).

**Figure 4.**
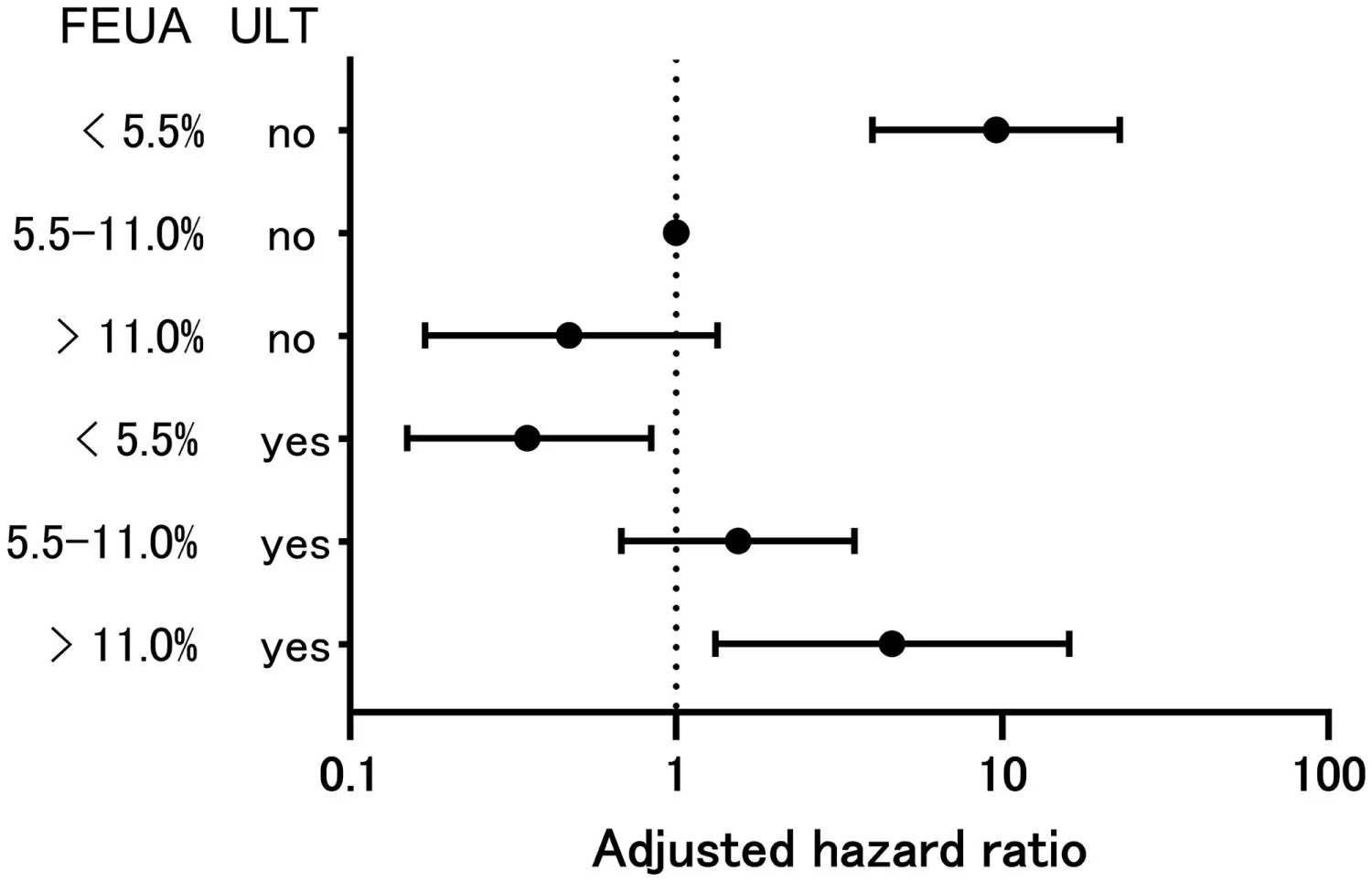
Effect modification by baseline ULT on the association between FEUA categories and composite outcome. Adjusted hazard ratios for the composite outcome for six subgroups defined based on FEUA category and baseline ULT use. The reference group comprised patients with FEUA of 5.5–11.0% not receiving ULT. Points indicate adjusted hazard ratios, and horizontal bars indicate 95% confidence intervals. Estimates were obtained from a multivariable Cox proportional hazards model including FEUA categories, ULT, and FEUA × ULT interaction terms, adjusted for age, sex, body mass index, baseline eGFR, systolic blood pressure, diabetes mellitus, proteinuria, urinary NAG–to–creatinine ratio, urinary β2MG–to–creatinine ratio, serum uric acid, serum albumin, and concomitant medications. ULT, urate–lowering therapy; FEUA, fractional excretion of uric acid.

To formally assess effect modification by baseline ULT use, we fitted a multivariable Cox model including FEUA categories, ULT, and FEUA × ULT interaction terms (Table 3 and Figure 4). Patients with FEUA of 5.5–11.0% without ULT served as the reference. In this model, the FEUA × ULT interaction was statistically significant (likelihood-ratio test p for interaction = 0.002). Relative to the reference group, the adjusted risk was markedly higher among untreated patients with FEUA <5.5% (adjusted HR, 9.60; 95% CI, 4.01–22.98). It did not differ significantly among untreated patients with FEUA >11.0% (adjusted HR, 0.47; 95% CI, 0.17–1.34). Among patients receiving ULT, the adjusted risk was lower in those with FEUA <5.5% (adjusted HR, 0.35; 95% CI, 0.15–0.84), but higher in those with FEUA >11.0% (adjusted HR, 4.60; 95% CI, 1.32–16.04). No significant difference was observed among ULT-treated patients with FEUA of 5.5–11.0% (adjusted HR, 1.55; 95% CI, 0.68–3.53). These findings indicate that the association between FEUA and the outcomes differed by ULT use, consistent with attenuation of the excess risk observed in the low FEUA group among those receiving ULT.

**Table 3.** Multivariable cox model including FEUA categories, ULT, and FEUA × ULT interaction terms.

FEUA	Baseline ULT	Adjusted HR (95% CI)	p value
< 5.5%	No	9.60 (4.01–22.98)	< 0.001
5.5-11.0%	No	Reference	―
> 11.0%	No	0.47 (0.17–1.34)	0.16
< 5.5%	Yes	0.35 (0.15–0.84)	0.018
5.5-11.0%	Yes	1.55 (0.68–3.53)	0.29
> 11.0%	Yes	4.60 (1.32–16.04)	0.016

P for interaction (FEUA × ULT): 0.002.

ULT, urate-lowering therapy; FEUA, fractional excretion of uric acid; HR, hazard ratio; CI, confidence interval.

To assess the robustness of the association between low FEUA and outcomes, we performed a sensitivity analysis in the no–ULT group. In this analysis, we excluded patients receiving medications known to influence tubular epithelial urate reabsorption. The direction and magnitude of the association for low FEUA were consistent with the primary analysis (Supplemental Fig. S3).

### Association between UUCR and kidney outcome

**Figure 5.**
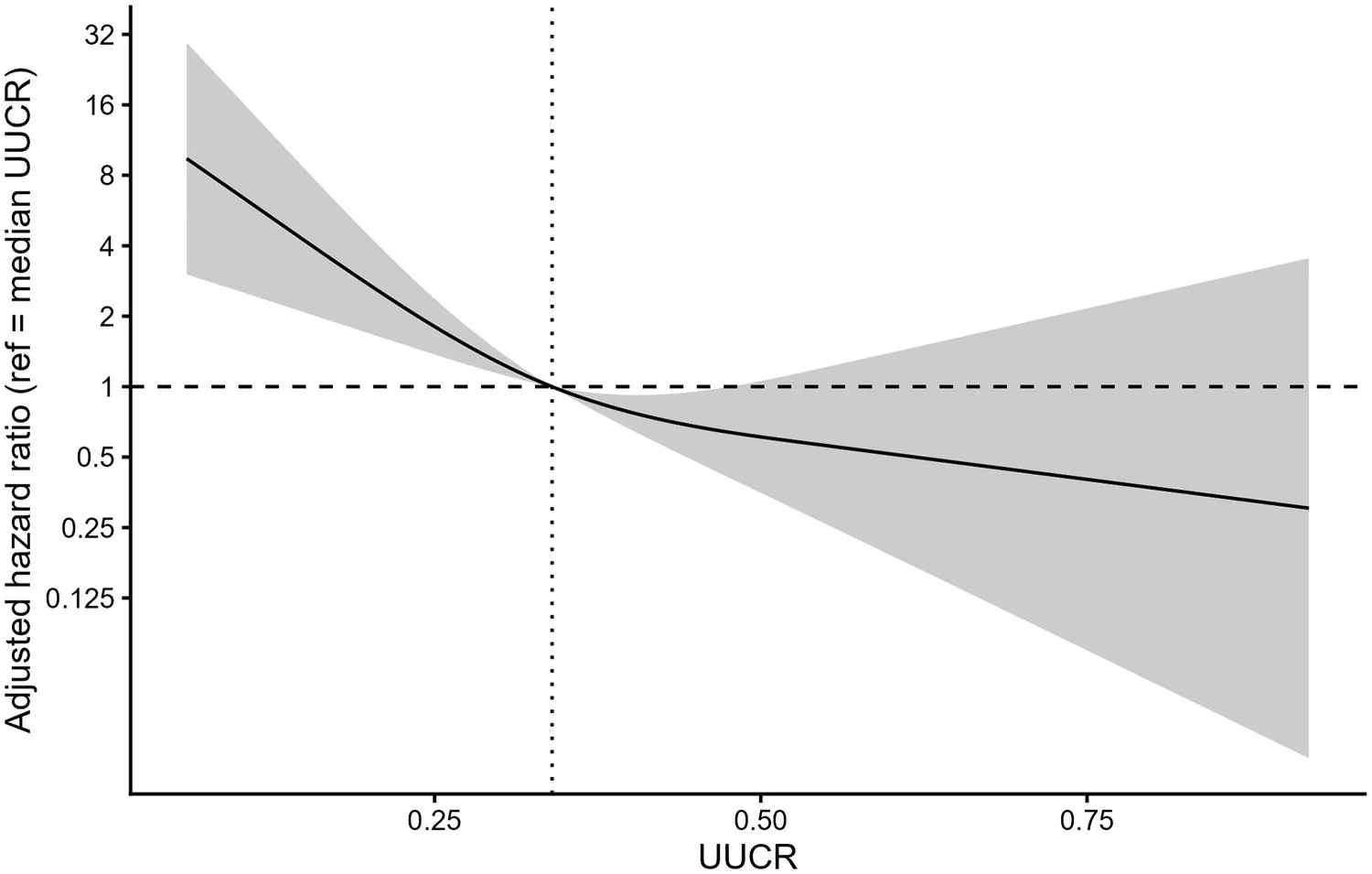
Restricted cubic spline analyses of UUCR in relation to outcome. Restricted cubic spline plots showing the adjusted association between UUCR (g/gCr) and the risk of the composite outcome. The solid line indicates the adjusted hazard ratio from the Cox model, and the shaded area indicates the 95% confidence interval. Hazard ratios are presented relative to the median value of each exposure (HR = 1, horizontal dashed line). Vertical dotted lines indicate the median exposure values. Models were adjusted for age, sex, body mass index, baseline eGFR, systolic blood pressure, diabetes mellitus, proteinuria, urinary NAG–to–creatinine ratio, urinary β2MG–to–creatinine ratio, serum uric acid, serum albumin, and concomitant medications, including urate-lowering therapy. UUCR, urinary uric acid–to–creatinine ratio.

In the secondary analyses, we examined the associations between UUCR and outcome. The restricted cubic spline analysis revealed an inverse association between UUCR and the risk of kidney disease. The adjusted hazard increased progressively as UUCR decreased below the median, indicating a steep risk gradient at lower UUCR levels (Figure 5).

**Figure 6.**
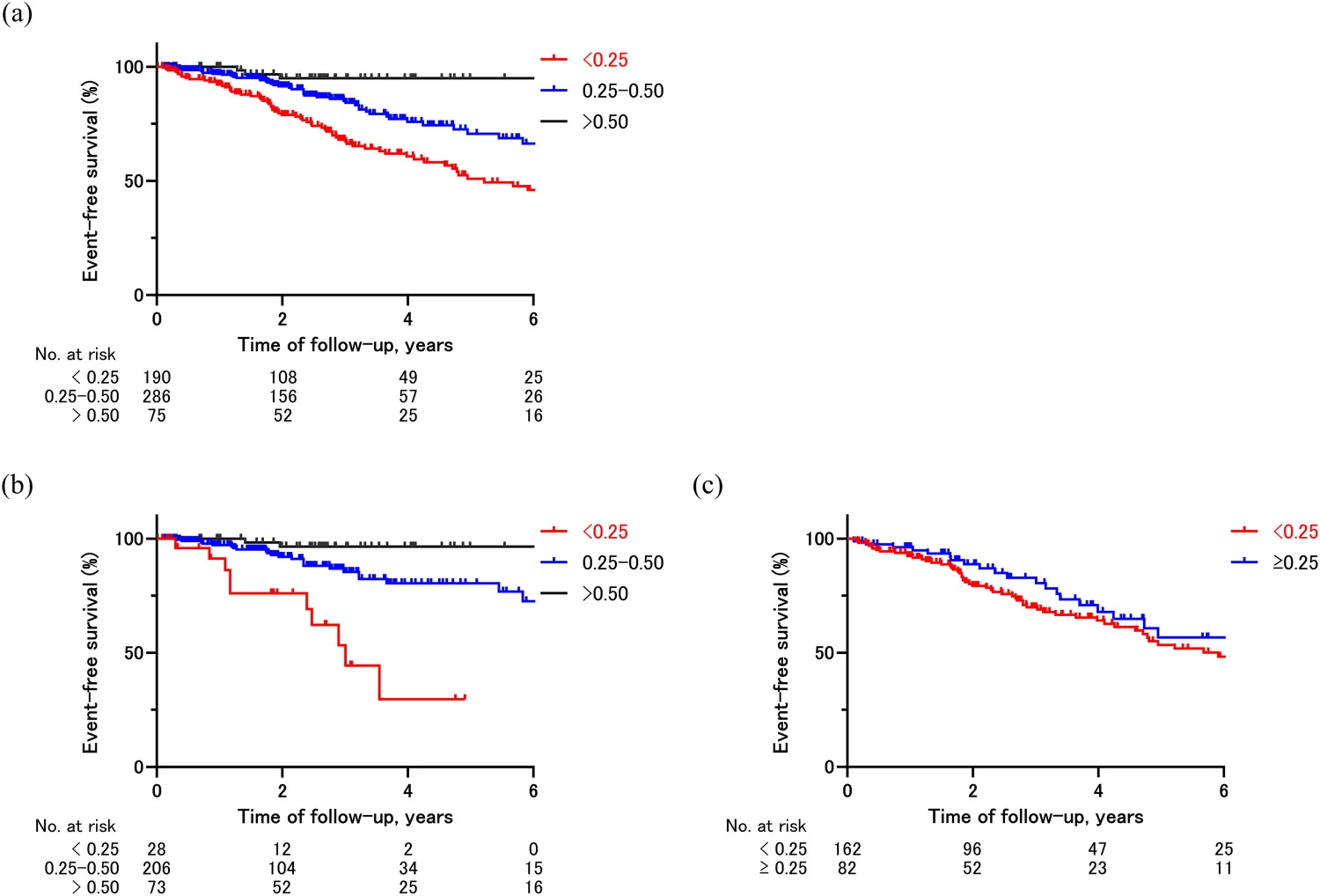
Kaplan–Meier curves for the composite kidney outcome by UUCR category. Kaplan–Meier curves for the composite kidney outcome stratified by baseline UUCR (g/gCr). (a) Overall cohort and (b) no-ULT group: UUCR categories <0.25, 0.25–0.50, and >0.50. (c) ULT group: categories were defined as <0.25 versus ≥0.25 because few patients treated with ULT had UUCR >0.50. Comparisons were performed using the log–rank test. ULT, urate-lowering therapy; UUCR, urinary uric acid-to-creatinine ratio.

Kaplan–Meier curves across UUCR categories are shown in Figure 6. In the overall cohort and in the no–ULT group, UUCR <0.25 g/gCr was associated with a higher incidence of kidney outcomes, whereas patients with UUCR > 0.50 g/gCr showed the most favorable kidney survival (log-rank *p* < 0.001 for both). In the ULT group, only two patients had UUCR > 0.50; therefore, UUCR ≥ 0.25 was analyzed as a single category. Kidney outcomes did not differ significantly between UUCR categories in the ULT group (log-rank *p* = 0.18).

In the multivariable Cox models adjusted for the same covariates as in the FEUA analyses (Table 4), UUCR < 0.25 g/gCr was significantly associated with a higher incidence of outcome in the overall cohort and in the no–ULT group (adjusted HR, 2.77; 95% CI, 1.52–5.05; *p* < 0.001 and 5.51; 95% CI, 1.89–16.12; *p* = 0.002, respectively). UUCR > 0.50 was not associated with kidney survival in the overall cohort and in the no–ULT group (adjusted HR, 0.47; 95% CI, 0.14–1.65; *p* = 0.24 and 0.30; 95% CI, 0.06–1.43; *p* = 0.13, respectively). In the ULT group, UUCR <0.25 vs. ≥0.25 was not associated with kidney outcome (adjusted HR, 1.59; 95% CI, 0.78–3.23; *p* = 0.20).

**Table 4.** Hazard ratios for kidney outcome according to UUCR categories.

Subgroup	UUCR category	Adjusted HR (95% CI)	*p* value
Overall	<0.25	2.77 (1.52–5.05)	<0.001
(*n* = 551)	0.25–0.50	Reference	–
	>0.5	0.47 (0.14–1.65)	0.24
No-ULT group	<0.25	5.51 (1.89–16.12)	0.002
(*n* = 307)	0.25–0.50	Reference	–
	>0.5	0.30 (0.06–1.43)	0.13
ULT group	≥0.25	Reference	–
(*n* = 244)	<0.25	1.59 (0.78–3.23)	0.20

ULT, urate lowering therapy; UUCR, urinary uric acid-to-creatinine ratio; HR, hazard ratio; CI, confidence interval.

## Discussion

In this study, reduced urinary uric acid excretion, as reflected by low FEUA and UUCR, was associated with a higher risk of the composite outcome in patients with CKD. Furthermore, baseline ULT modified these associations. Low FEUA and UUCR were strongly associated with a higher risk in patients not receiving ULT, even after adjustment for baseline eGFR, serum uric acid, and conventional risk factors. In contrast, no clear independent association was observed among those receiving ULT. The significant FEUA × ULT interaction indicates that the prognostic meaning of FEUA depends on concomitant ULT.

The overall association we observed is consistent with a previous CKD cohort study, in which lower FEUA and UUCR were associated with a higher risk of kidney failure [29]. However, we found that when stratified by ULT use, the association was clearly concentrated in the no–ULT group, with no significant association observed within the ULT group. These results indicate that pooling treated and untreated patients may obscure clinically relevant heterogeneity and support the use of ULT as an important effect modifier when interpreting urinary urate indices.

FEUA and UUCR were strongly correlated; however, they capture complementary aspects of urate handling. UUCR, a spot urine index of urinary urate excretion relative to creatinine, has been proposed as a practical surrogate for urinary urate load, and a threshold of around 0.5 g/gCr may help identify underexcretion phenotypes [30–32]. In contrast, FEUA reflects the excreted fraction of the filtered urate load [22,33]. ULT reduces serum and urinary urate excretion by lowering filtered urate load, while FEUA may remain relatively stable [33–35]. Nevertheless, the significant interaction in our study suggests that the clinical meaning of a given FEUA differs by ULT status. The excess risk associated with low FEUA in untreated patients was attenuated among patients receiving ULT. By contrast, the interaction model showed a higher adjusted risk in ULT-treated patients with high FEUA than in untreated patients with normal FEUA, although high FEUA was not independently associated with the outcome within the ULT group. Patients receiving ULT had lower baseline eGFR and different comorbidity and medication profiles, and those with high FEUA within this group had the lowest eGFR. Therefore, the higher risk observed in ULT-treated patients with high FEUA likely reflects advanced kidney dysfunction rather than an independent effect of high FEUA. A high FEUA in advanced CKD may also represent compensatory urate excretion by the remaining functioning nephrons under increased physiological demand, although this interpretation remains speculative. These observations support the possibility that ULT may mitigate risk associated with reduced urinary urate excretion.

Our findings are consistent with experimental and clinical evidence implicating tubular urate exposure in kidney injury [15]. Urate transport into the proximal tubular cells can promote oxidative stress and inflammatory signaling [16–18,36]. Because FEUA reflects urinary urate as a fraction of the filtered load, a low FEUA itself indicates enhanced net tubular reabsorption or reduced secretion, rather than reduced glomerular filtration. A greater urate uptake into proximal tubular cells would increase intracellular urate exposure even when less urate is ultimately excreted in the urine. Therefore, a low FEUA is interpreted as a marker of increased intracellular urate handling rather than of diminished urate delivery to the nephron. Clinical studies in advanced CKD also suggested that urinary urate indices are associated with biomarkers of tubular injury and fibrosis [37,38]. Collectively with these data, our findings support a pathophysiological model in which reduced urinary urate excretion may reflect a tubular phenotype that is more susceptible to injury and subsequent progression of CKD [15].

This study has some limitations. Given that this was a single-center, retrospective observational study, residual confounding by unmeasured factors, including dietary habits, genetic variants affecting urate transporters, and indications for ULT, cannot be excluded. FEUA and UUCR were calculated from single-spot urine samples, and very high UUCR values were uncommon, particularly in the ULT group, limiting precision and the upper range. In addition, we could not evaluate differences in ULT regimen between XOIs and uricosuric agents.

In conclusion, reduced urinary uric acid excretion, as reflected by low FEUA and UUCR, was associated with an increased risk of kidney disease progression, particularly among patients not receiving ULT at baseline. The significant interaction between FEUA and ULT use indicates that the prognostic meaning of FEUA differs by ULT status. The excess risk linked to low FEUA in untreated patients was not evident in those receiving ULT, whereas a high FEUA in ULT-treated patients was associated with higher risk in interaction analysis, likely reflecting advanced kidney dysfunction and compensatory urate excretion by the remaining functioning nephrons. These results highlight the clinical relevance of intrarenal urate handling in CKD progression and suggest that urinary urate indices may help identify high-risk patients when interpreted together with ULT status and kidney function. Prospective studies and randomized trials are needed to determine whether targeting urate handling patterns can improve kidney outcomes in patients with CKD.

## Supplementary Material

Supplementary_Materials_R1.docx

## Data Availability

The data presented in this study are available upon reasonable request from the corresponding author.
