# Supplementary material for: Urate-lowering therapy modifies associations between urinary urate and kidney outcome
Source: Ren Fail. 2026 Aug 24;48(1):2700810. doi: 10.1080/0886022X.2026.2700810 (PMC13508531; doi:10.1080/0886022X.2026.2700810)
Supplement: Supplementary file 1 [file IRNF_A_2700810_SM7460.docx]

**SUPPLEMENTARY MATERIALS**

**Table of contents**

**Supplemental Tables**

- Table S1. Baseline characteristics of patients not receiving ULT.
- Table S2. Baseline characteristics of patients receiving ULT.

**Supplemental Figures**

- Fig. S1. Baseline urinary urate indices based on ULT status.
- Fig. S2. Correlations between urinary urate indices and kidney function.
- Fig. S3. Sensitivity analyses excluding medications that may influence tubular urate handling in the no-ULT group.

| Supplemental Table S1. Baseline characteristics in patients not receiving ULT | | | | |  |  |
| --- | --- | --- | --- | --- | --- | --- |
|  | FEUA (%) | | |  | |  |
|  | < 5.5 | 5.5–11.0 | > 11.0 | p value | |  |
| Number | 45 | 194 | 68 |  | |  |
| Age, years | 67 ± 15 | 70 ± 13 | 69 ± 12 | 0.51 | |  |
| Male, n (%) | 26 (58%) | 112 (58%) | 48 (63%) | 0.72 | |  |
| BMI, kg/m^2^ | 24.0 ± 4.3 | 23.9 ± 3.9 | 23.3 ± 3.7 | 0.47 | |  |
| Systolic blood pressure, mmHg | 135 ± 21 | 140 ± 23 | 137 ± 26 | 0.32 | |  |
| Diastolic blood pressure, mmHg | 79 ± 13 | 81 ± 14 | 80 ± 17 | 0.73 | |  |
| Diabetes mellitus, n (%) | 13 (29%) | 63 (33%) | 23 (34%) | 0.85 | |  |
| Hypertension, n (%) | 33 (73%) | 140 (72%) | 52 (77%) | 0.79 | |  |
| Cardiovascular disease, n (%) | 11 (24%) | 44 (23%) | 18 (27%) | 0.81 | |  |
| Gout, n (%) | 1 (2%) | 1 (1%) | 0 (0%) | 0.33 | |  |
| Loop diuretics, n (%) | 8 (18%) | 20 (10%) | 3 (4%) | 0.069 | |  |
| Thiazide diuretics, n (%) | 5 (11%) | 15 (8%) | 2 (3%) | 0.23 | |  |
| RAS blockades, n (%) | 28 (62%) | 100 (52%) | 35 (52%) | 0.41 | |  |
| Statins, n (%) | 21 (47%) | 61 (31%) | 31 (46%) | 0.038 | |  |
| SGLT2 inhibitors, n (%) | 0 (0%) | 12 (6%) | 4 (6%) | 0.23 | |  |
| Albumin, g/dL | 3.9 ± 0.5 | 3.9 ± 0.6 | 3.7 ± 0.7 | 0.12 | |  |
| Creatinine, mg/dL | 1.1 (1.0-1.4) | 1.2 (1.1-1.4) | 1.5 (1.2-2.1) | <0.001 | |  |
| eGFR, mL/min/1.73m^2^ | 44.1 ± 10.2 | 41.9 ± 10.8 | 33.8 ± 11.6 | <0.001 | |  |
| Uric acid, mg/dL | 7.5 ± 1.9 | 6.6 ± 1.3 | 5.8 ± 1.5 | <0.001 | |  |
| C–reactive protein, mg/dL | 0.2 (0.1-0.6) | 0.1 (0.0-0.3) | 0.1 (0.0-0.3) | 0.11 | |  |
| Aciduria, n (%) | 23 (51%) | 81 (42%) | 15 (22%) | 0.003 | |  |
| UPCR, g/gCr | 0.3 (0.1-1.5) | 0.3 (0.1-1.3) | 0.8 (0.2-2.0) | 0.016 | |  |
| Urinary NAG–to–Cr ratio, U/gCr | 11.7 (7.2-19.1) | 10.1 (5.7-14.8) | 13.8 (7.7-20.2) | 0.047 | |  |
| Urinary β2MG–to–Cr ratio, µg/gCr | 196 (80-561) | 426 (175-1881) | 4855 (1188-18984) | <0.001 | |  |
| FEUA, % | 4.4 (3.8-5.2) | 7.8 (6.9-9.2) | 12.9 (11.6-16.0) | <0.001 | |  |
| UUCR, g/gCr | 0.28 (0.21-0.33) | 0.43 (0.35-0.49) | 0.49 (0.40-0.63) | <0.001 | |  |
| BMI, body mass index; RAS, renin–angiotensin system; sodium–glucose cotransporter; eGFR, estimated glomerular filtration rate; UPCR, urinary protein–to–creatinine ratio; NAG, N-acetylglucosamine; β2MG, beta 2–microglobulin; FEUA, fractional excretion of uric acid; UUCR, urinary uric acid–to–creatinine ratio | | | | |  |  |
|  |  |  |  |  |  | |
|  |  |  |  |  |  | |

| Supplemental Table S2. Baseline characteristics in patients receiving ULT | | | | |  |
| --- | --- | --- | --- | --- | --- |
|  | FEUA (%) | | |  |  |
|  | < 5.5 | 5.5–11.0 | > 11.0 | p value |  |
| Number | 129 | 102 | 13 |  |  |
| Age, years | 69 ± 13 | 71 ± 12 | 70 ± 13 | 0.27 |  |
| Male, n (%) | 103 (80%) | 77 (76%) | 11 (85%) | 0.62 |  |
| BMI, kg/m^2^ | 23.7 ± 3.8 | 24.5 ± 4.4 | 26.3 ± 4.7 | 0.051 |  |
| Systolic blood pressure, mmHg | 129 ± 21 | 139 ± 22 | 147 ± 29 | <0.001 |  |
| Diastolic blood pressure, mmHg | 75 ± 15 | 79 ± 16 | 81 ± 18 | 0.16 |  |
| Diabetes mellitus, n (%) | 24 (19%) | 34 (33%) | 3 (23%) | 0.037 |  |
| Hypertension, n (%) | 110 (85%) | 92 (90%) | 11 (85%) | 0.51 |  |
| Cardiovascular disease, n (%) | 45 (35%) | 38 (37%) | 7 (54%) | 0.40 |  |
| Gout, n (%) | 8 (6%) | 7 (7%) | 1 (8%) | 0.97 |  |
| Loop diuretics, n (%) | 32 (25%) | 20 (20%) | 2 (15%) | 0.53 |  |
| Thiazide diuretics, n (%) | 13 (10%) | 14 (14%) | 2 (15%) | 0.060 |  |
| RAS blockades, n (%) | 78 (61%) | 71 (70%) | 11 (85%) | 0.13 |  |
| Statins, n (%) | 59 (46%) | 45 (44%) | 4 (31%) | 0.066 |  |
| SGLT2 inhibitors, n (%) | 4 (3%) | 4 (4%) | 1 (8%) | 0.055 |  |
| Albumin, g/dL | 3.9 ± 0.5 | 3.9 ± 0.6 | 3.7 ± 0.5 | 0.25 |  |
| Creatinine, mg/dL | 1.5 (1.3-1.9) | 1.8 (1.3-2.2) | 2.4 (1.9-2.6) | <0.001 |  |
| eGFR, mL/min/1.73m^2^ | 34.9 ± 11.3 | 31.1 ± 11.4 | 23.8 ± 7.5 | <0.001 |  |
| Uric acid, mg/dL | 6.5 ± 1.3 | 6.3 ± 1.6 | 5.2 ± 1.1 | 0.008 |  |
| C–reactive protein, mg/dL | 0.2 (0.1-0.5) | 0.1 (0.1-0.3) | 0.1 (0.1-0.3) | 0.52 |  |
| Aciduria, n (%) | 71 (55%) | 41 (40%) | 2 (15%) | 0.022 |  |
| UPCR, g/gCr | 0.3 (0.1-0.9) | 0.5 (0.1-2.0) | 1.8 (1.0-2.8) | <0.001 |  |
| Urinary NAG–to–Cr ratio, U/gCr | 9.4 (5.4-14.7) | 11.6 (6.9-18.4) | 18.7 (13.1-24.9) | 0.004 |  |
| Urinary β2MG–to–Cr ratio, µg/gCr | 134 (57-723) | 2103 (335-10417) | 17397 (5055-45877) | <0.001 |  |
| FEUA, % | 4.1 (3.3-4.8) | 7.3 (6.1-8.6) | 14.4 (11.7-14.4) | <0.001 |  |
| UUCR, g/gCr | 0.16 (0.12-0.20) | 0.26 (0.20-0.34) | 0.32 (0.29-0.36) | <0.001 |  |
| BMI, body mass index; RAS, renin–angiotensin system; sodium–glucose cotransporter; eGFR, estimated glomerular filtration rate; UPCR, urinary protein–to–creatinine ratio; FEUA, fractional excretion of uric acid; NAG, N-acetylglucosamine; β2MG, beta 2–microglobulin; UUCR, urinary uric acid–to–creatinine ratio; ULT, urate–lowering theapy; XOI, xanthine oxidase inhibitor | | | | |  |
|  |  |  |  |  |  |
|  |  |  |  |  |  |
|  |  |  |  |  |  |

**Supplemental Figure S1. Baseline urinary urate indices based on ULT status.** Distributions of baseline (a) FEUA and (b) UUCR in patients not receiving urate-lowering therapy (no-ULT group) and those receiving urate-lowering therapy (ULT group). Each dot represents an individual patient; summary bars indicate medians with interquartile ranges. ULT, urate-lowering therapy; FEUA, fractional excretion of uric acid; UUCR, urinary uric acid–to–creatinine ratio. ***, p < 0.001.

**Supplemental Figure S2. Correlations between urinary urate indices and kidney function.** Scatter plots showing correlations between (A) FEUA and UUCR and (B) FEUA and eGFR in the overall cohort. Solid lines represent fitted linear regression lines (Pearson correlation: FEUA vs UUCR, r = 0.708; FEUA vs eGFR, r = −0.113). FEUA, fractional excretion of uric acid; UUCR, urinary uric acid–to–creatinine ratio; eGFR, estimated glomerular filtration ratio.

**Supplemental Figure S3. Sensitivity analyses excluding medications that may influence tubular urate handling in the no-ULT group.** Adjusted hazard ratios (HRs) for the composite kidney outcome associated with low FEUA (<5.5%) compared with the reference (FEUA 5.5–11.0%) in the no-ULT group after excluding patients receiving medications potentially affecting tubular urate transport. Exclusion sets comprised diuretics (loop or thiazide), uricosuric angiotensin II receptor blockers (irbesartan or losartan), SGLT2 inhibitors, atorvastatin, fenofibrate, and all of the above. “No” indicates the primary analysis without medication exclusions. Points indicate adjusted HRs, and horizontal bars indicate 95% confidence intervals. Models were adjusted for the covariates specified in the Methods. ULT, urate–lowering therapy; FEUA, fractional excretion of uric acid; SGLT2, sodium–glucose cotransporter 2.
